# Environmental key performance indicators: the role of regulations and stakeholder influence

**DOI:** 10.1007/s10669-021-09825-z

**Published:** 2021-07-23

**Authors:** Ewelina Zarzycka, Joanna Krasodomska

**Affiliations:** 1 University of Lodz, Lodz, Poland; 2 Cracow University of Economics, Cracow, Poland

**Keywords:** Key performance indicators, Stakeholders, Regulations, Environment

## Abstract

Environmental protection is of vital importance and needs to be considered in the context of business strategies, including companies’ reporting decisions. This paper aims to investigate the importance of stakeholders for environmental key performance indicators (KPIs) and the significance of different types of environmental KPIs to various stakeholders. The study is based on a content analysis of the disclosures provided by large public interest companies operating in Poland. The data were processed to produce descriptive statistics as well as classification and regression trees (C&RTs). According to the study results, the sample companies provide a variety of environmental indicators, with a total of 735 KPIs identified. The research confirms the importance of stakeholders interested in environmental issues for corporate decisions regarding environmental KPI disclosure. The study contributes to the extant literature by providing new insights into the importance of different stakeholder groups for the disclosure of environmental KPIs. It may serve as an incentive for standard setters and practitioners to take a proactive approach in further developing and improving environment-related reporting regulations.

## Introduction

According to a United Nations report (UN 2020), the natural environment continues to deteriorate at an alarming rate. Observable high water stress, unsustainable use of resources, inadequate waste management, a slow increase in the share of renewable energy in total energy consumption, and still high greenhouse gas (GHG) emissions could have devastating consequences for the environment and our common future. Therefore, environmental issues require special attention (Collier et al. 2021).

The COVID-19 outbreak, apart from its obvious negative consequences, has led to some positive environmental impacts such as enhanced air and water quality in urban areas, mainly due to massive decreases in transportation usage and industrial activities (Cheval et al. 2020). However, scientists warn that even this sharp but relatively brief decline is unlikely to make a meaningful difference in the long run if governments do not make environmental protection a priority (Lewis 2020).

Although responsibility for environmental protection is considered to lie primarily with countries and governments, the role of business in this process is critical. Governments do not have enough resources to provide all the solutions necessary to solve such problems. Therefore, the public and private sectors need to cooperate, and businesses need to contribute to the more sustainable global economy by undertaking initiatives aimed at environmental protection. Relevant policy- and decision-making frameworks are needed to improve the actual situation and enable evaluation of the results of the environmental actions undertaken (Linkov et al. 2020).

Although the proper choice of metrics is crucial for progress on environmental protection and restoration programs as well as for management decision making related to those programs (Convertino et al. 2013), it is still a challenge to effectively report and measure these initiatives (UN 2020). There is no single, uniform methodology for measuring and reporting business impacts on the environment. Apart from the Global Reporting Initiative (GRI) Standards, several initiatives have been launched to develop an agreed-upon and harmonized set of indicators for consistent and comparable sustainability reporting. In this study, we concentrate on European Union (EU) initiatives, with a particular focus on the Directive 2014/95/EU (EU 2014), the European Commission’s (EC) guidelines (EC 2017, 2019) and the use of key performance indicators (KPIs) as a tool to measure corporate environmental strategy and its impacts. Despite the acknowledged effort made by the directive to increase the comparability of nonfinancial disclosures across Europe, it is said not to be as effective as assumed in regard to meeting stakeholders’ information needs (Szabó and Sørensen 2015; EC 2020; Pizzi et al. 2020; Venturelli et al. 2019, 2020).

As stakeholders demand more information about companies’ environmental performance, firms need to become more proactive in their measurement and reporting of practices (Perego and Hartmann 2009; Burnett and Hansen 2008). According to Burritt and Schaltegger (2010), companies should consult with different stakeholders to obtain agreement on appropriate KPIs for measuring environmental practice. Prior studies document a positive evolution in the quantity of environmental information reported as a response to the pressure exerted by stakeholders (Isaacs et al. 2015; Moneva and Llena 2000). Stakeholders are perceived as influencing the firm’s choice of KPIs for measuring social practice through environmental strategies (mediated influence), direct influence on KPI selection, joint efforts whereby stakeholders and firms interact to achieve common environmental goals, and social and environmental performance benchmarking (cf. Lisi 2018; Rodrigue et al. 2013). Thus, the identification of stakeholders and characterization of organizational relationships with each group are essential, as stakeholders should assist organizations in developing and improving environmental strategies and related KPI disclosures (Kaur and Lodhia 2018).

With this paper, we intend to highlight the importance of stakeholders for environmental KPI disclosures and to investigate what types of such disclosures are significant for various stakeholders. We formulate the following research questions: *Which stakeholder groups influence corporate strategy with regard to environmental KPI disclosure? What categories of KPIs are the most significant for stakeholders?*

The study is based on a content analysis of the disclosures provided by 169 large public interest entities operating in Poland in 2019. Further, the data were processed using descriptive statistics and classification and regression trees (C&RTs). The study findings indicate that the sample companies provide a variety of environmental indicators. The research confirms the importance of environmental pressure groups and stakeholders interested in environmental issues for disclosing KPIs related to environmental matters. This finding is in line with earlier studies that state that environment-related issues are disclosed by companies operating in environmentally sensitive industries, in which stakeholders' awareness of environmental issues is significant (Fernandez-Feijoo et al. 2014; Gamerschlag et al. 2011; Banerjee et al. 2003). The research identifies the most important groups of KPIs for customers, investors and employees.

This research is intended to make three important contributions to the extant literature and practice. First, in a broader sense, it provides new insights into environmental KPI disclosures, thus, contributing to the literature on corporate social responsibility (CSR) (cf. Lisi 2018; Nielsen et al. 2017; Chang et al. 2014; Arvidsson 2011; Andrew and Cortese 2011). Second, it adds a missing element to stakeholder theory, as it focuses particularly on the importance to stakeholders of the abovementioned disclosures. Despite stakeholders' environmental concerns, there has been scant evidence on their influence on KPIs for environmental practice (Thomson 2007). Thus, our study fills that gap by addressing the importance of different environmental KPIs for various stakeholders (Lisi 2018; Fernandez-Feijoo et al. 2014). Third, given that environmental disclosures attract special attention from regulators and other institutions, the study may be interesting for governmental agencies, national accounting associations, standard setters, and practitioners to further develop and improve the European Commission’s *Guidelines on reporting climate-related information* issued in 2019 (EC 2019).

The work is structured into four sections. The next section describes EU initiatives which focus on corporate environment-related reporting, more specifically Directive 2014/95/EU and subsequent EC guidelines. The section that follows provides information on the theoretical background of the study and presents the research question. After that, the empirical research methods and findings are discussed. The last section offers conclusions together with an indication of potential avenues for future research.

## Environment-related nonfinancial disclosures within the European Union reporting landscape

In October 2014, the EU adopted Directive 2014/95/EU. The directive aimed to ‘increase the relevance, consistency, and comparability of information disclosed by certain large undertakings and groups across the Union’ (EU 2014, Introduction par. 21). Starting in 2018, approximately 6000 large public interest entities (PIEs^1^) in the EU, with an average of 500 employees or more, began to disclose nonfinancial information about their practices concerning environmental matters, social and employee-related issues, respect for human rights, and anticorruption and bribery. Nonfinancial disclosures can be presented in the management commentary or in a separate report. They include a description of the company's business model, nonfinancial policies, and nonfinancial KPIs.

According to Directive 2014/95/EU (EU 2014, Introduction par. 7), regarding environmental matters, a nonfinancial disclosure should contain ‘details of the current and foreseeable impacts of the undertaking's operations on the environment, and, as appropriate, on health and safety, the use of renewable and/or nonrenewable energy, greenhouse gas emissions, water use and air pollution.’ To provide disclosures, companies can use various national, EU-based, and international recommendations.

To help companies provide high-quality, relevant, consistent and more comparable nonfinancial (environmental, social and governance-related) information that will be useful to stakeholders, the EC has published two sets of guidelines: *Guidelines on non-financial reporting (methodology for reporting non-financial information)* (EC 2017) and *Guidelines on reporting climate-related information. Guidelines on non-financial reporting: Supplement on reporting climate-related information* (EC 2019).

The latter set of EC guidelines focuses on climate change and is based on the Task Force on Climate-related Financial Disclosures (TCFD^2^) Recommendations (EC 2019). In addition to the TCFD, EC guidelines also take particular account of the standards and frameworks developed by the Global Reporting Initiative (GRI), the CDP, the Climate Disclosure Standards Board (CDSB), the Sustainability Accounting Standards Board (SASB) and the International Integrated Reporting Council (IIRC) and of the EU Eco-Management and Audit Scheme (EMAS).

Like the general guidelines published in 2017, the 2019 supplement on climate-related reporting is nonbinding. The EC recognizes that the content of climate-related disclosures may vary between companies according to several factors, including the sector of activity. The guidelines provide a flexible approach but encourage companies to integrate climate-related information with other financial and nonfinancial information rather than offer stand-alone nonfinancial reporting. The TCFD proposes that its recommended disclosures be included in the company’s mainstream ‘annual financial filings.’ Companies should also seek to ensure that climate-related information is easily accessible to stakeholders (EC 2019).

The EC defines KPIs as indicators that are ‘consistent with metrics actually used by the company in its internal management and risk assessment process’ (EC 2017, p. 13). They represent a set of measures focusing on the factors most critical for the success of an organization now and in the future (Parmenter 2015). The use of KPIs in corporate reporting should improve the transparency and comparability of disclosures. Thus, KPIs should be broadly recognized, material, useful, relevant, and of high quality. Bakkensen et al. (2017) underline that the indicators should clearly focus on stating their objective and require prior testing to ensure that they perform well. According to Directive 2014/95/EU, companies should disclose KPIs relevant to their particular business. They ought to consider using indicators to support their other climate-related disclosures, such as those related to outcomes or principal risks and their management, and allow for aggregation and comparability across companies and jurisdictions. Indicators should be integrated with other disclosures to support and explain the narrative. However, it is also considered good practice to publish an additional table that presents all KPIs in one place. To meet the expectations of the TCFD, companies should disclose KPIs and targets used by the company to assess climate-related risks and opportunities in line with their strategy and risk management processes. The robustness and reliability of data are key to enabling the use of the information in decision-making processes. Where not obvious, companies should provide a description of and name any changes in the methodologies used to calculate or estimate the KPIs. Table 1 presents the KPIs that, according to the EC guidelines, companies should consider disclosing to facilitate greater comparability of disclosures of nonfinancial information (EC 2019).

**Table 1:** Environmental KPIs proposed by the EC guidelines

KPI [unit of measurement]	Rationale
GHG emissions	
Direct GHG emissions from sources owned or controlled by the company (Scope 1) [tons]	Measuring carbon footprints from direct emissions
Indirect GHG emissions from the generation of acquired and consumed electricity, steam, heat, or cooling (collectively referred to as ‘electricity’) (Scope 2) [tons]	Measuring emissions from purchased or acquired electricity, steam, heat, and cooling
All indirect GHG emissions (not included in scope 2) that occur in the value chain of the reporting company, including both upstream and downstream emissions (Scope 3) [tons]	Capturing the thoroughness of companies’ accounting processes and understanding how companies analyze their emissions footprints. For most companies, the majority of emissions occur indirectly from value chain activities
GHG absolute emissions target [tons or %]	Measuring companies' commitments to reducing emissions and assessing whether they have a goal toward which they are harmonizing and focusing emissions-related efforts
Energy	
Total energy consumption [MWh] and/or production from renewable and nonrenewable sources [MWh]	Measuring energy consumption and production, which account for an important proportion of GHG emissions
Energy efficiency target [%]	Capturing the companies’ ambition to use energy more efficiently, which can reduce their energy costs and lower GHG emissions. This KPI provides further background on how the company aims to achieve its emissions reduction targets
Renewable energy consumption [% increase in the proportion of renewable energy consumed] and/or production target [% increase in the proportion of renewable energy produced from base year]	Measuring the companies’ ambition to produce or consume energy with lower GHG emissions
Physical risks	
Assets committed in regions likely to become more exposed to acute or chronic physical climate risks [%]	Capturing interruptions to or limitations on production capacity or early curtailment of operating facilities due to extreme weather events. The value of assets in areas exposed to volatile weather offers information on the potential implications for asset valuation. It is important to observe this KPI in conjunction with disclosures regarding the company’s adaptation strategies and policies
Products and services	
Percent turnover in the reporting year from products or services associated with activities that meet the criteria for substantially contributing to mitigation of or adaptation to climate change as set out in the regulation on the establishment of a framework to facilitate sustainable investment (EU taxonomy) [%] and/or Percent investment (CapEx) and/or expenditures (OpEx) in the reporting year for assets or processes associated with activities that meet the criteria for substantially contributing to mitigation of or adaptation to climate change as set out in the regulation on the establishment of a framework to facilitate sustainable investment (EU taxonomy) [%]	Capturing how company’s products and services substantially contribute to mitigation of or adaptation to climate change while not significantly harming any of the EU’s other environmental objectives
Green finance	
Climate-related green bond ratio: Total amount of green bonds outstanding (at year end) divided by (a 5-year rolling average of) the total amount of bonds outstanding [%] and/or Climate-related green debt ratio: Total amount of all green debt instruments outstanding (at year end) divided by (a 5-year rolling average of) the total amount of all debt outstanding [%]	Capturing how companies’ low-carbon transition plan is supported by debt financing activities and how capital is raised for existing and new projects with climate benefits

EC guidelines propose 14 KPIs, which refer to five areas: GHG emissions, energy consumption, physical risk, products and services, and green finance. Apart from measuring companies’ negative and common environmental impacts, such as GHG emissions or energy use, the proposed indicators also capture the direct relation between environmental protection and corporate financial performance, e.g., KPIs related to green finance (the climate-related green bond ratio or climate-related green debt ratio).

## Study design

### Conceptual framework and research question

Corporate decisions regarding environmental disclosure can be examined through the lens of stakeholder theory (Freeman 1984). According to this theory, the relationship between the company and stakeholders generates a need for the company to accommodate the interests and needs of its stakeholders with regard to environmental protection (Solikhah et al. 2021). Companies’ managers carry out strategies to accomplish their responsibilities to stakeholders; nonfinancial disclosure, in turn, is a form of corporate responsibility to society and a way of fulfilling the information needs of investors, employees, customers, ecologists, and other parties.

Continuous environmental degradation has triggered increased stakeholder pressure on companies to change their behavior and focus more on their environmental impacts (Kitsikopoulos et al. 2018; Helfaya and Moussa 2017; Isaacs et al. 2015; Johnson et al. 2013; Hovardas and Poirazidis 2007). Prior research has analyzed stakeholder pressures and corporate responses (Sprengel and Busch 2010) with regard to environmental strategies (Seroka-Stolka and Fijorek 2020). Many of these works recognize stakeholder engagement as the core principle to enhance accountability and transparency in regard to environmental and social issues (see Aureli et al. 2020; Kaur and Lodhia 2018; Rinaldi et al. 2014; Gao and Zhang 2001). First, investors seem to have a considerable influence on companies’ actions. Fewer and fewer investors are short sighted, and there is a growing number of socially responsible investors. They take environmental and social issues into account in their investment decisions in addition to financial considerations (Eurosif 2018). De Villers (2018), Cubas-Díaz and Martínez Sedano (2017), and Rinaldi et al. (2014) provide evidence that investors not only use sustainability information in their investment decisions but may also demand such disclosures from companies. Second, when employees see that they and other stakeholders are treated fairly through social practices, they may also engage in such social practices within the companies (Huang and Kung 2010; Cropanzano and Rupp 2008). Third, a number of studies indicate a positive relationship between a company’s CSR actions and consumers’ purchase intentions and attitudes toward that company (see, among others, Auger et al. 2003; Collison et al. 2003). Although there are studies that suggest that customers have a positive impact on carbon disclosure, research supporting this association is still scarce (Hahn et al. 2015). Finally, companies under pressure from environmental pressure groups and ecological activists disclose environmental information to a larger extent than those that are not exposed to such influences (Hahn et al. 2015; Gamerschlag et al. 2011).

In this study, we investigate corporate disclosure practices in the context of Directive 2014/95/EU. The directive has changed the nonfinancial reporting landscape in Europe. It is often referred to as a document that has introduced mandatory nonfinancial reporting in EU member states (cf. Cordazzo et al. 2020; Caputo et al. 2020; Matuszak and Różańska 2017). However, the directive and the guidelines that followed give companies a great deal of flexibility on what and where to disclose and which nonfinancial reporting framework to use. Moreover, the directive uses the ‘comply or explain’ approach and is principles based. The flexibility of a ‘comply or explain’ approach to disclosure also makes it a particularly well-suited strategy for nonfinancial reporting, which is, to a large extent, driven by the industry in which a company operates and its specific characteristics. The principles-based disclosure strategy also fits better with the nature of nonfinancial information than prescriptive one-size-fits-all reporting rules (Ho 2017). In a situation where there is considerable flexibility regarding corporate reporting decisions, stakeholder engagement is seen as a crucial element of effective corporate communication. Therefore, we expect to be able to identify the influence of stakeholder groups on corporate reporting practices with respect to KPI disclosures and to investigate the significance of different types of environmental KPIs to various stakeholders, contributing to the further development of stakeholder theory. This study aims to answer the following research questions: *Which stakeholder groups influence corporate strategy with regard to environmental KPI disclosure? What categories of KPIs are the most significant for stakeholders?*

### Sample, data collection, and research method

In this study, we investigate the reporting practices of 169 large PIEs based in Poland and required to provide nonfinancial disclosures according to the Polish Act on Accounting, which includes the Directive 2014/95/EU requirements. Our sample covers all entities that fell under the directive’s scope in Poland during the sample period. We accessed the sample companies’ nonfinancial statements published in 2019 via the website https://standardy.org.pl/raporty-spolek (FSR 2020) in PDF format and performed a content analysis of the disclosures on nonfinancial KPIs related to environmental matters. Content analysis is widely used in disclosure studies as a method of collecting data (Guthrie 2014; Beattie and Thomson 2007). To identify the KPIs, a word search was performed with the following words and phrases in a source file (usually a PDF): ‘KPI,’ ‘key,’ and ‘indicator’ (both in English and in Polish). The information on KPIs included in a nonfinancial statement was transferred to an observation sheet and coded. KPIs related to the environment were divided into the following main areas: (1) emissions, (2) water use, (3) wastewater, (4) raw material consumption, (5) energy use, (9) packaging and waste, and (7) environmental investments, infractions, controls, and fines. A simple binary (0,1) coding scheme was used to indicate the presence or absence of an item in each category. The coding procedure was performed by the coauthors. Intercoder reliability was tested with the use of Krippendorf’s alpha (Lombard et al. 2002).

Next, the environmental KPI disclosure index (ENV_KPI) was calculated as the ratio of all environmental KPIs disclosed by the company to the maximum number of environmental KPIs. The index for the abovementioned seven categories of environmental KPIs was calculated for every company as the ratio of the number of KPIs presented by the company in a given category to the maximum number of KPIs presented in this category. Thus, the total environmental KPI disclosure index and seven indexes for the indicated categories are calculated using the following formula:

$${\text{ENV}}\_{\text{KPI}}_{i} = \frac{{\sum {{\text{KPI}}_{i} } }}{{{\text{MAX}}\_{\text{KPI}}}},$$

where: ENV_KPI_*i*_—environmental KPIs disclosure index for company *i, *KPI_*i*_—number of environmental KPIs presented by company *i, *MAX_KPI—maximum number of environmental KPIs presented.

In summary, the environmental KPI disclosure index (ENV_KPI) comprises all environmental KPIs presented by the companies, while the seven index components are based on the KPIs disclosed in the identified categories. The approach used falls into the category of disclosure index studies (Beattie et al. 2004).

Previous studies have found a significant relationship between company nonfinancial disclosures and industry (Pérez et al. 2015; Gamerschlag et al. 2011; Ho and Taylor, 2007) as well as between such disclosures and specific stakeholder group pressures (Krasodomska and Zarzycka 2020; Bradley and Botchway 2018; Pérez et al. 2015; Fernandez-Feijoo et al. 2014; Chang et al. 2014; Arvidsson 2011; Adams et al. 1998) and preferences (Convertino et al. 2013). The connection between the two stems from the fact that the specifics of companies’ operations, determined by the industry in which they operate, attract the interests of various stakeholders, and make the influence of certain groups more important than that of others (cf. Pérez et al. 2015; Fernandez-Feijoo et al. 2014; Adams et al. 1998).

Thus, following Fernandez-Feijoo et al. (2014), we identified the stakeholders of the company (customers, employees, environmental pressure groups, and investors) according to an industry categorization that reflects the presence or absence of stakeholder influence (Table 2). Actual and potential shareholders represent the primary stakeholders in investor-oriented industries (IOI) (Collins 2010; Sweeney and Coughlan 2008). These are industries in which more than 50% of companies are traded on the stock exchange. According to the literature, the automotive, aviation, chemicals, computers, conglomerates, construction, construction materials, consumer durables, energy, energy utilities, financial services, healthcare products, household and personal products, media, metals products, real estate, retailers, technology hardware, telecommunications, textiles and apparel, and toys industries are investor oriented (cf. Fernandez-Feijoo et al. 2014; Collins 2010; Sweeney and Coughlan 2008).

**Table 2:** Variable descriptions

Variable	Description	Measurement	References
ENV_KPI	Environmental KPIs disclosed by companies	Ratio of the number of all environmental KPIs disclosed by the company to the maximum number of environmental KPIs	EC (2019), Beattie et al. (2004)
Emissions	KPIs regarding emissions disclosed by companies	Ratio of the number of KPIs regarding emissions presented by the company to the maximum number of KPIs presented in this category	EC (2019), Beattie et al. (2004)
Water use	KPIs regarding water use disclosed by companies	Ratio of the number of KPIs regarding water use presented by the company to the maximum number of KPIs presented in this category	EC (2019), Beattie et al. (2004)
Wastewater	KPIs regarding wastewater disclosed by companies	Ratio of the number of KPIs regarding wastewater presented by the company to the maximum number of KPIs presented in this category	EC (2019), Beattie et al. (2004)
Raw material consumption	KPIs regarding raw material consumption disclosed by companies	Ratio of the number of KPIs regarding raw material consumption presented by the company to the maximum number of KPIs presented in this category	EC (2019), Beattie et al. (2004)
Energy use	KPIs regarding energy use disclosed by companies	Ratio of the number of KPIs regarding energy use presented by the company to the maximum number of KPIs presented in this category	EC (2019), Beattie et al. (2004)
Packaging and waste	KPIs regarding packaging and waste disclosed by companies	Ratio of the number of KPIs regarding packaging and waste presented by the company to the maximum number of KPIs presented in this category	EC (2019), Beattie et al. (2004)
Environmental investments, infractions, controls, and fines	KPIs regarding environmental investments, infractions, controls, and fines disclosed by companies	Ratio of the number of KPIs regarding environmental investments, infractions, controls, and fines presented by the company to the maximum number of KPIs presented in this category	EC (2019), Beattie et al. (2004)
IOI	Investor-oriented industry: main stakeholders—investors	1—company belongs to an investor-oriented industry such as the automotive, aviation, chemicals, computers, conglomerates, construction, construction materials, consumer durables, energy, energy utilities, financial services, healthcare products, household and personal products, media, metals products, real estate, retailers, technology hardware, telecommunications, textiles and apparel and toys industries; 0—other industry	Fernandez-Feijoo et al. (2014), Collins (2010), Sweeney and Coughlan (2008)
EOI	Employee-oriented industry: main stakeholders—employees	1—company belongs to an employee-oriented industry and employs more than 1949 employees (median); 0—other industry	Fernandez-Feijoo et al. (2014), Haski-Leventhal (2013), Aldama et al. (2009), Ellis (2009), Wei et al. (2009)
COI	Customer-oriented industry: main stakeholders—customers	1—company belongs to a consumer-oriented industry such as the energy utilities, financial services, food and beverage products, healthcare, textiles and apparel, retailers, household and personal products, telecommunications, waste management, water utilities, commercial services, consumer durables, media, tobacco, tourism/leisure, toys, and universities industries; 0—other industry	Fernandez-Feijoo et al. (2014), Sweeney and Coughlan (2008), Branco and Rodrigues (2008)
*EUI*	Industries with a negative impact on the environment: main stakeholders—environmental pressure groups	1—company belongs to the industries with a negative impact on the environment such as the agriculture, automotive, aviation, chemical, construction, construction materials, energy, energy utilities, forest and paper products, logistics, metal products, mining, railroad, waste management, and water utilities industries; 0—other industry	Fernandez-Feijoo et al. (2014), Gamerschlag et al. (2011), Tagesson et al. (2009), Branco and Rodrigues (2008)

In customer-oriented industries (COI), which are well known to members of the general public, as consumers of these industries’ products or services, customers play a major role (Fernandez-Feijoo et al. 2014; Sweeney and Coughlan 2008; Branco and Rodrigues 2008). Accordingly, we identified the following industries as customer oriented: energy utilities, financial services, food and beverage products, healthcare, textiles and apparel, retailers, household and personal products, telecommunications, waste management, water utilities, commercial services, consumer durables, media, tobacco, tourism/leisure, toys, and universities.

Industries that have a negative impact on the environment (EUI), such as the agriculture, automotive, aviation, chemical, construction, construction materials, energy, energy utilities, forest and paper products, logistics, metal products, mining, railroad, waste management, and water utilities industries, are under pressure from ecological groups (Fernandez-Feijoo et al. 2014; Gamerschlag et al. 2011; Sweeney and Coughlan 2008; Tagesson et al. 2009; Branco and Rodrigues 2008).

Apart from the industry types presented, we also distinguish employee-oriented industries (EOI), which are identified based on the size of a company. We assume that large and multinational companies experience more pressure from employees, as they are better organized and their opinions are taken into consideration by managers (Fernandez-Feijoo et al. 2014; Haski-Leventhal 2013; Aldama et al. 2009; Ellis 2009; Wei et al. 2009).

As previously mentioned, company stakeholders were identified following Fernandez-Feijoo et al. (2014) based on the relationship between the main stakeholder groups and the industries. Industry data were collected from the companies’ reports. Twenty-four different sectors were identified, and in line with Fernandez-Feijoo et al. (2014), four variables were created considering the pressure on each industry from four groups of stakeholders (customers, employees, environmental pressure groups, and investors). Table 2 presents the description of the variables used, together with the information on how they were measured and relevant sources.

The data were processed using descriptive statistics and C&RTs. C&RT analysis is a recursive partitioning method that builds classification and regression trees for predicting dependent variables (regression) and predictor variables (classification). Moreover, this technique enables the construction of variable importance charts, which determine which predictors are the most important for the classification trees. The importance is defined as the percent improvement with respect to the most important predictor that is used as the primary splitter in the tree. The variable with the highest improvement score is set as the most important variable, while the other variables are ranked accordingly. Importance, in turn, is calculated by dividing each variable importance score by the largest importance score of the variables and then multiplying it by 100%. C&RTs are particularly useful for data mining tasks and reveal relationships between variables that could pass unnoticed with the use of other traditional analytical tools (see Ripley 1996; Breiman et al. 1984).

## Results

### Descriptive statistics

Table 3 presents the details of the industries covered by the study. The most widely represented are investor-oriented companies (65% of the researched companies). Half of the companies belong to employee- and customer-oriented industries. Finally, 48% of the entities under research are classified as environmentally unfriendly companies.

**Table 3:** Sample companies according to type

Type of company	% of companies
Industries with a negative impact on the environment (EUI)	48
Employee-oriented companies (EOI)	50
Customer-oriented companies (COI)	50
Investor-oriented companies (IOI)	65

The companies included in the sample present a total of 154 different environmental KPIs referring to seven categories: packaging and waste; energy use; environmental investments, infractions, controls, and fines; raw material consumption; emissions; water use; and wastewater (Table 4). “Appendix” contains examples of the KPIs in each category for three selected companies from different industries.

**Table 4:** Details concerning the identified environmental KPIs

KPIs	No. of KPIs	No. of observations	Mean
Packaging and waste	57	216	1.172
Energy use	26	133	0.787
Environmental investments, infractions, fines	25	55	0.355
Raw material consumption	21	75	0.444
Emissions	10	147	0.941
Water use	9	76	0.450
Wastewater	6	33	0.195

The total number of observations is 735 (Table 4). The most frequently presented KPIs are those related to packaging and waste. The use of energy is presented according to the energy source: electricity, fuels, and natural gases. These KPIs are expressed not only in natural units but also in relation to revenue or to the number of clients. Some companies present an ‘energy efficiency’ ratio or an ‘intensiveness of energy use’ ratio. The next KPI group is very diverse, as it comprises important aspects related to environmental issues such as environmental investments, infractions, controls, and fines. Of these, the most often presented KPIs are associated with the number of environmental fines. KPIs related to raw material consumption report the quantity or value of the most important materials used in the companies in total or in relation to revenues. In the case of emissions, the amount of carbon dioxide is most frequently presented, followed by information on other selected substances. Again, these KPIs are usually expressed in natural units. However, some companies also show the amount of emissions in relation to the unit of production or revenues. KPIs on the use of water are expressed most often in natural units. Only a few companies present indicators of water use in relation to the number of employees, units of production or revenues. Finally, KPIs reporting on waste are most frequently divided into two groups—hazardous and nonhazardous—and according to how they are utilized. KPIs are usually expressed in tons but also in relation to the total amount of waste to the unit of production or revenue.

In a comparison of the environmental KPIs disclosed by the sample companies with those proposed in the EC guidelines (EC 2019), it is noticeable that they do not cover all suggested areas and are presented more simplistically than is recommended by the EC. GHG emissions and energy use KPIs are widely used, but no KPIs related directly to, e.g., green finance and presented in the way proposed by the EC are identified. At the same time, KPIs referred to, e.g., water use and wastewater, often reported by the sample companies, are not included in the EC guidelines.

### C&RT analysis

#### The importance of stakeholder influence in the corporate decision to disclose environmental KPIs: General overview

**Fig. 1:**
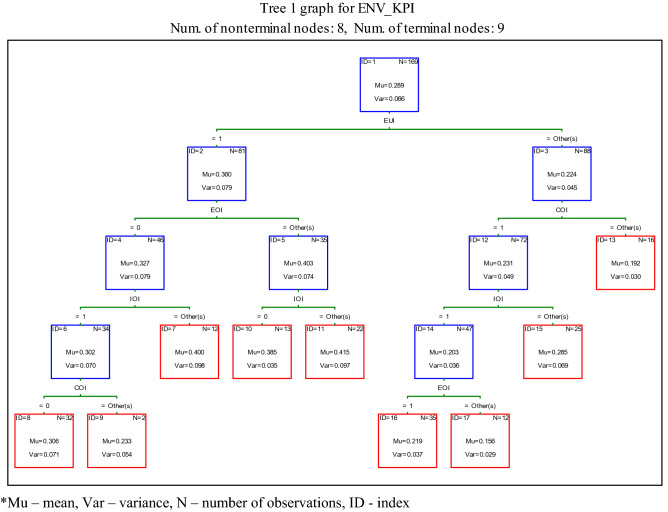
Tree for the average number of environmental KPIs disclosed by companies under the influence of different stakeholders

To generally determine the importance of stakeholders' influence on the corporate decision to disclose environmental KPIs, classification and regression trees (C&RTs) are used. The tree graph for the classification tree on the ratio of environmental KPIs disclosed by companies (ENV_KPI) under the influence of different stakeholders is shown in Fig. 1. The classification tree has 9 terminal nodes. In the graph, terminal nodes are outlined with red lines, while the remaining split nodes are outlined with blue lines. The tree graph shows that companies from environmentally unfriendly industries and under the influence of employees and investors disclose, on average, more environmental KPIs (node ID = 24, Mu = 0.415). Interestingly, the companies representing unfriendly industries, despite being less influenced by other stakeholders, report more on environmental issues. For example, facing pressure from only two groups of stakeholders, ecologists and employees, results in a relatively high intensity of disclosure (node ID = 10, Mu = 0.385). Similarly, in the absence of influence from employees but under pressure from investors, unfriendly industries present a higher number of environmental KPIs (node ID = 7, Mu = 0.400). It is clear that companies not facing pressure from ecologists, other environmentally concerned stakeholders or customers disclose on average fewer environmental KPIs (node ID = 24; Mu = 0.192). Surprisingly, the lowest level of environmental disclosure is observable among companies not influenced by environmental pressure groups and employees but rather by customers and investors (node ID = 17; Mu = 0.156).

**Fig. 2:**
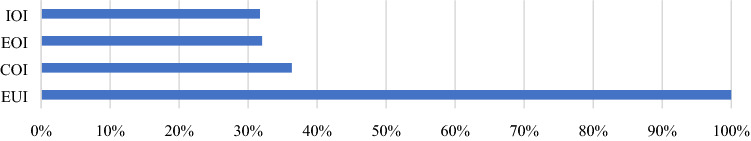
The importance of stakeholders for the corporate decision to disclose environmental KPIs (%)

When univariate splits are performed, the predictor variables can be ranked on a 0–100 scale in terms of their potential importance in accounting for responses of the dependent variable (see Breiman et al. 1984). For the corporate decision to provide environmental KPI disclosures, environmental pressure groups’ (EUI) information needs are clearly very important, while employees (EOI) are relatively unimportant (see Fig. 2).

#### The importance of identified categories of environmental KPIs to different stakeholders

**Fig. 3:**
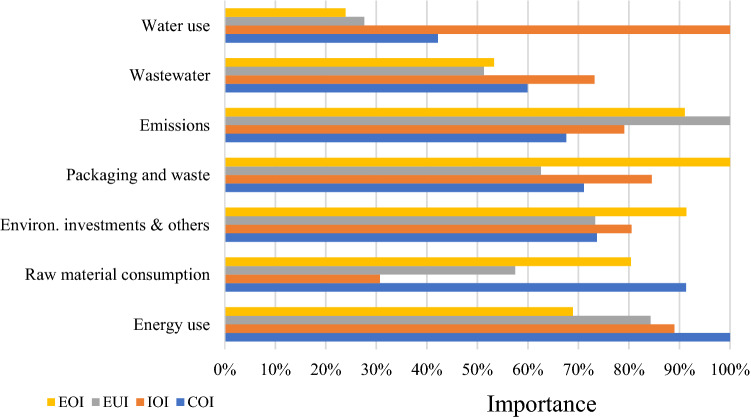
The importance of identified categories of environmental KPIs to stakeholders (%)

Similar analyses are performed to determine the importance to different stakeholder groups of the seven categories of KPIs: water use; wastewater; emissions; packaging and waste; environmental investments, infractions, controls, fines and others; raw material consumption; and energy use (Fig. 3).

KPIs regarding water use are clearly very important for investors (IOI) and relatively unimportant for employees (EOI). Similarly, KPIs on wastewater have significant meaning for investors (IOI), while the importance for other stakeholder groups is at a similar but slightly lower level. It is worth noting that wastewater indicators are of relatively minor importance for all groups. This lower interest may result from the fact that water use and wastewater are not included in the EC guidelines (EC 2019).

The emissions category of KPIs appears to be one of the most important in general. These indicators are clearly very important not only for environmental pressure groups (EUI), but also for employees (EOI) and investors (IOI). The importance of these indicators may stem from the need to harmonize and focus on emissions-related efforts, as underlined by the EC guidelines (EC 2019) as well as the TCFD recommendations (TCFD 2020). Similarly, KPIs regarding energy use are relatively of high importance for almost all stakeholder groups, mainly for customers (COI), investors (IOI) and environmental pressure groups (EUI). This category of KPIs, alongside that of GHG emissions, most commonly measures companies’ negative environmental impact and represents the KPI groups indicated in the EC guidelines (EC 2019).

KPIs on packaging and waste as well as environmental investments, infractions, controls, and fines are important for employees (EOI) and investors (IOI). Finally, raw material consumption KPIs are the most important for customers (COI) and employees (IOI) and relatively unimportant for investors (IOI).

In summary, the study confirms the importance of ecologists and stakeholders interested in environmental issues as pressure groups for disclosing KPIs related to environmental issues in general. In particular, KPIs on GHG emissions and energy use are of high importance for this group of stakeholders. This finding is in line with earlier research showing that environment-related KPIs are widely disclosed by companies operating in environmentally sensitive industries, in which stakeholders’ awareness of environmental issues is significant (cf. Fernandez-Feijoo et al. 2014; Gamerschlag et al. 2011; Tagesson et al. 2009; Branco and Rodrigues 2008).

Moreover, the results reveal the importance of selected environmental KPIs for customers, investors, and employees (cf. Fernandez-Feijoo et al. 2014; Branco and Rodrigues 2008; Sweeney and Coughlan 2008). We assume that investors’ interest in packaging and waste, energy use and water use, and wastewater KPIs stems from the impact of the mentioned categories on the financial performance of the company. Investors are seen as important users of nonfinancial information provided by companies (De Villiers 2018), and environmental disclosures are useful for their decision making and investments (cf. Cubas-Díaz and Martínez Sedano 2017; Rinaldi et al. 2014). Interestingly, customers find the energy use and raw material consumption KPIs to be the most important. Energy use is a commonly used indicator of companies’ negative environmental impacts (EC 2019), while indicators of raw material consumption provide customers with information on the sustainability of material sources used for production purposes and, thus, may influence their purchase intentions and attitudes toward the company (see, among others, Auger et al. 2003; Collison et al. 2003).

According to Huang and Kung (2010) and Fernandez-Feijoo et al. (2014), employees generally impact sustainability reporting and the level of environmental disclosure. Our study also reveals the wide interest of this group in the various environmental KPIs. Table 5 summarizes the importance of the KPIs for different stakeholder groups.

**Table 5:** The importance of importance of KPIs for different stakeholder groups

KPI category	Most important stakeholders
Environmental—in general	Environmental pressure groups
Packaging and waste	Employees, investors
Energy use	Customers, investors, environmental pressure groups
Environmental investments & others	Employees, investors
Raw material consumption	Customers, employees
Emissions	Environmental pressure groups, employees, investors
Water use	Investors
Wastewater	Investors

## Conclusions

Directive 2014/95/EU certainly changed the status of corporate nonfinancial reporting in the EU. However, it seems that by choosing the significantly cheaper minimum harmonization approach, not supported by detailed rules and standards on the collection and processing of information, the EC’s action, in the end, did not produce the intended effects (Szabó and Sørensen 2015). As noted in EC communication (EC 2020), market pressures on their own have not proven to be sufficient to ensure that companies report the nonfinancial information that stakeholders say that they need. According to several studies, the main effects of Directive 95/2014/EU have been limited to an increase in the overall number of nonfinancial reports disclosed each year and not necessarily the quality of the provided disclosures (Pizzi et al. 2020; Venturelli et al. 2019, 2020). Therefore, a revision of the EU Directive is expected and should seek to ensure that corporate disclosures more effectively communicate their social and environmental impacts and—as a part of this process—support the development of EU sustainability reporting standards (EFRAG 2021).

Regarding environmental protection, the EU has agreed on ambitious targets for 2030 regarding GHG emission reductions, renewable energy, and energy efficiency and has approved rules on GHG emissions from land use as well as emission targets for cars and vans. In 2018, the commission published its long-term strategic vision for a prosperous, modern, competitive, and climate-neutral economy by 2050. The EU acknowledges the critical role that companies and financial institutions have to play in the transition to a more sustainable economy (EC 2019). An effective measurement and reporting system built on the proper KPIs can be crucial for measuring progress toward this goal.

The literature related to corporate environmental discourse is extensive (Andrew and Cortese 2011); however, few studies focus specifically on environmental KPI disclosures. Nonfinancial KPIs in general have been investigated in relation to, e.g., their use by managers for internal purposes, their impact on corporate performance, differences in KPI disclosure regulations and practices, and stakeholder pressure to provide disclosures (e.g., Krasodomska and Zarzycka, 2020; Bradley and Botchway 2018; Lisi 2018; Nielsen et al. 2017; Chang et al. 2014; Arvidsson 2011). Nevertheless, none of these studies examine environmental KPI disclosures in more detail. Therefore, the current study aims to fill this research gap by providing new insights into this important area of nonfinancial KPI disclosures in a new and relatively unexplored institutional setting.

According to our research findings, 169 large PIEs operating in Poland disclose 154 different environmental KPIs in a diverse manner, considering that the total number of observations is equal to 735. Our findings show that large PIEs operating in Poland most widely report KPIs related to packaging and waste, GHG emissions, and energy use. Out of these KPIs, emissions are reported in the most comparable way.

We also find that the influence of environmental pressure groups seeking information related to corporate performance in this regard is the primary driver of environment-related KPI disclosure. The information needs of other stakeholder groups, such as customers, employees, and investors, were identified with respect to specific environmental KPIs. These findings support the results of previous studies (Gamerschlag et al. 2011; Tagesson et al. 2009; Branco and Rodrigues 2008; Sweeney and Coughlan 2008).

Our study findings add to the CSR literature, as we can confirm that the results of previous studies on the influence of the industry and stakeholders on corporate nonfinancial disclosures also hold for environmental KPI disclosures. They also provide new insights into the role of regulation and stakeholder influence in the reporting process. We believe that effective KPI reporting should balance the two. The important change in Polish accounting law introduced by Directive 2014/95/EU provided an incentive to companies to disclose environmental KPIs. Given the flexibility of the regulation, companies’ managers must take into consideration stakeholders' information needs in their disclosure decision-making processes, and our study shows that they do so. Without responding to these needs, companies’ KPI disclosures would be too complex and less comparable, and their usefulness would decrease.

Our study is also relevant for practice. KPI selection and disclosures are more subject to management decisions, as they are used both internally and for external reporting purposes. The incentives created by the regulatory framework to present particular environmental KPIs and supported by the results of a dialog between companies and environmentally engaged stakeholder groups can trigger companies to start to measure and monitor these KPIs strategically. Therefore, companies that are externally obliged to disclose KPIs may introduce internal changes in their practices. Subsequently, these decisions may allow them to operate more effectively and sustainably. It also seems that to adhere to the EC guidelines (EC 2019), the sample companies should reconsider their KPI selection and construction. For example, the KPI *Percent investment (CapEx) and/or expenditures (OpEx) in the reporting year for assets or processes associated with activities that meet the criteria for substantially contributing to mitigation of or adaptation to climate change as set out in the regulation on the establishment of a framework to facilitate sustainable investment (EU taxonomy)*, not reported by these firms so far, might require more effort to provide relevant information than the effort needed to report on, e.g., direct GHG emissions.

The findings clearly show that stakeholders drive companies’ strategy with regard to KPI disclosure. Therefore, possible new nonfinancial EU-wide reporting standards have to be established in a dialogic process with various stakeholder groups. For the regulation of environmental disclosures, not only environmental pressure groups but also customers', employees’, and investors’ opinions are important. Joint collaboration of governmental agencies, regulatory bodies, businesses, and stakeholders is needed to take action that could change corporate reporting practices and contribute to a sustainable future. Without a transparent approach to the use of KPIs for the measurement of companies’ progress toward this goal, country-level efforts might also be hard to evaluate and manage effectively.

The study is not free from limitations. We cover the whole population of large PIEs operating in Poland. However, their number is limited to 169 entities. We also focus on only one country—Poland—and analyze data for only 1 year—2019. Additionally, content analysis, which we use as our main research approach, is not free from limitations, such as subjectivity, but we took all measures to minimize them, including a test of intercoder reliability.

The findings of this study invite researchers to further explore environmental KPI disclosures. Replication of the study with more data from different countries could enable the generalization of the study results. Furthermore, an interesting direction for future research would be to explore concrete situations regarding stakeholders’ interactions with companies’ managers on the development of environmental KPIs. Such case studies could focus especially on the information needs of selected stakeholders. Their findings might shed more light on the interactions between the regulatory framework, stakeholder engagement, and managers’ strategic decisions. Studies to help managers translate regulations and stakeholder expectations into a tool that managers could implement while preparing disclosures might also be useful. Finally, additional opportunities for future research can be found in relation to addressing the impact of COVID-19 on corporate environmental disclosures.

## Appendix: Examples of KPIs disclosed by sample companies from different industries

**Table:**

Category of KPIs	Disclosed KPIs
Company 1: Construction industry	
Emissions	Amount of carbon dioxide produced Amount of emissions in relation to the unit of production or revenues Total amount of ozone-depleting substances (ODSs) by net value added
Water use	Use of water in natural units
Wastewater	Wastewater in natural units
Raw material consumption	Use of selected materials to units of production
Energy use	Use of energy in natural units
Packaging and waste	Quantity of hazardous waste in natural units Nonhazardous waste produced in absolute terms Proportion of hazardous waste treated, given total waste reported by the reporting entity in absolute amounts, in % terms and in terms of change Change in the entity’s waste generation by net value added in % terms, in terms of change and in absolute amounts
Environmental investments, infractions, fines	No disclosures
Company 2: Automotive industry	
Category of KPIs	Disclosed KPIs
Emissions	Scope 1,2,3
Water use	Use of water to units of production Use of water to revenues
Wastewater	Wastewater to units of production Total volume of water recycled and/or reused during the reporting period in absolute amounts
Raw material consumption	Use of selected materials in natural units Use of selected materials to revenues
Energy use	Energy expenses to revenues Use of energy in natural units
Packaging and waste	Quantity of hazardous waste in natural units
Environmental investments, infractions, fines	The number of fines related to the environment Value of fines related to the environment
Company 3: Financial services	
Category of KPIs:	Disclosed KPIs
Emissions	Amount of carbon dioxide produced by company cars
Water use	Water use in relation to the number of employees
Wastewater	Wastewater to revenues
Raw material consumption	No disclosures
Energy use	energy efficiency ratio
Packaging and waste	The entity’s waste generation in terms of change and in absolute amounts
Environmental investments, infractions, fines	No disclosures

## Footnotes

1 PIEs are entities governed by the law of an EU member state whose transferable securities are traded on a regulated market of any member state, credit institutions, insurance undertakings, or entities designated by member states as such. Examples of PIEs include public companies, banks, or insurers (Deloitte 2015).

2 The TCDF was established in 2015 by the Financial Stability Board at the request of G20 finance ministers and central bank governors. The TCDF’s 32 international members, led by Michael Bloomberg, include providers of capital, insurers, large nonfinancial companies, accounting and consulting firms, and credit rating agencies. The TCFD recommendations, established in June 2017, are ‘designed to solicit consistent, decision-useful, forward-looking information on the material financial impacts of climate-related risks and opportunities, including those related to the global transition to a lower-carbon economy’ (TCFD 2020). They are widely recognized as authoritative guidance on the reporting of financially material climate-related information and implemented into the national frameworks of several countries, including the EU member states.
